# Prevalence and Predictors of Insulin Resistance in Non-Obese Healthy Young Females in Qatar

**DOI:** 10.3390/ijerph17145088

**Published:** 2020-07-15

**Authors:** Mohamed A. Elrayess, Nasser M. Rizk, Amina S. Fadel, Abdelhamid Kerkadi

**Affiliations:** 1 Biomedical Research Center, Qatar University, Doha 2713, Qatar; 2 Department of Biomedical Science, College of Health Sciences, QU-Health, Qatar University, Doha 2713, Qatar; nassrizk@qu.edu.qa (N.M.R.); aminafadel@qu.edu.qa (A.S.F.); 3 Physiology Department, Mansoura Faculty of Medicine, Mansoura University, Mansoura 35516, Egypt; 4 Human Nutrition Department, College of Health Science, QU-Health, Qatar University, Doha 2713, Qatar

**Keywords:** non-obese, insulin resistance, prevalence, BMI, Qatar

## Abstract

The state of Qatar suffers from diabetes epidemic due to obesity-associated metabolic syndrome. However, the prevalence of insulin resistance prior to obesity, which could play an important role in the high prevalence of diabetes, has not yet been described. This study aims to compare the prevalence of insulin resistance in apparently healthy non-obese and obese participants from Qatar and identify the predictors of insulin resistance in different body mass index (BMI)-groups. In this cross-sectional study, 150 young healthy females from Qatar were dichotomized into four groups (underweight, normal weight, overweight and obese) based on their BMI. Anthropometric measures as well as fasting plasma levels of lipids, adipokines, blood glucose and insulin were recorded. The prevalence of insulin resistance as per homeostatic model assessment of insulin resistance (HOMA-IR) was estimated and differences between insulin sensitive and insulin resistant were compared. Linear models were used to identify predictors of insulin resistance in every BMI group. Prevalence of insulin resistance in non-obese healthy females from Qatar ranges between 7% and 37% and increases with BMI. Overall, predictors of insulin resistance in the Qatari population are triglycerides/high-density lipoprotein (HDL) ratio and free fat mass but vary according to the BMI group. The main predictors were triglycerides in normal weight, triglycerides/HDL in overweight and triglycerides/HDL and interleukin-6 (IL-6) in obese individuals. The high prevalence of insulin resistance in non-obese Qataris may partially explain diabetes epidemic. Larger studies are warranted to confirm these findings and identify underlying causes for insulin resistance in non-obese individuals in Qatar, aiming at targeted intervention before diabetes onset.

## 1. Introduction

The global prevalence of diabetes among adults of 20–79 years of age has risen from 4.7% in 1980 to 8.8% (95% confidence interval 7.2%–11.3%) in 2017 [1]. The epidemiologic transition to sedentary lifestyle has contributed significantly to this epidemic, especially in the developing countries and among certain ethnicities [2]. Qatar is a clear example of such a transition as a healthy life style (pearl hunting and sea food-diet) has dramatically changed in the past 5 decades as the country’s economy has become mostly dependent on gas and oil [3,4]. The overall prevalence of type II diabetes (T2D) among adult Qatari population is currently alarmingly high (23.3%) with greater propensity among women [5].

The association between insulin resistance and obesity is well established [6], however various studies have shown that individuals with normal body weight can also become insulin resistant (IR) and, if left untreated, may eventually develop T2D and cardiovascular disease despite being non-obese [7,8,9]. Available evidence suggests that people with a body mass index (BMI) between 20 and 27 kg/m^2^ who have gained 2–10 kg of fat mass during adulthood constitute perfect candidates to develop insulin resistance and subsequently T2D with risk factors including central fat distribution, inactivity and a low maximum oxygen uptake (VO2max) [9]. Since these factors are potentially reversible by controlling diet, exercise and possibly through pharmacological intervention, understanding the pathophysiology of insulin resistance in non-obese individuals may offer a better chance for treating insulin resistance before development of T2D with potentially irreversible consequences. Studies have shown that long-term (5–6 years) diet and exercise can diminish T2D incidence in lean/overweight subjects with impaired glucose tolerance [9]. Additionally, lean-IR individuals tend to be younger as obesity increases with age; hence they are potentially more responsive to diet and exercise than their older obese counterparts. Based on this evidence, targeted therapies aimed at young lean individuals with insulin resistance could prevent the development of T2D and other diseases, including perhaps obesity itself.

In order to improve the identification of insulin resistance in healthy individuals, many studies have investigated the utility of various surrogate markers of insulin resistance. One study compared the use of triglycerides/glucose against triglyceride/high-density lipoprotein (TG/HDL) cholesterol ratio as potential surrogate markers. The study identified that both estimates correlated with steady-state plasma glucose concentration to a similar degree with comparable associations to estimates using fasting insulin [10]. Another study set to evaluate the association between TG/HDL ratio and insulin resistance or hyperinsulinemia after oral glucose tolerance test (OGTT) in normal-weight healthy adults. The study showed that high TG/HDL ratio was indeed associated with both insulin resistance markers [11]. Similarly, the association between elevated triglycerides/glucose index (TGI) and insulin resistance or hyperinsulinemia after oral glucose tolerance test (OGTT) revealed that elevated TGI was associated with insulin resistance in healthy adults. The study suggested that the simplicity of the TGI calculation makes it the first alternative when the hyperinsulinemic–euglycemic clamp or homeostatic model assessment of insulin resistance (HOMA-IR) are not available [12].

Despite the high prevalence of diabetes in the Qatari population, the prevalence of insulin resistance among non-obese apparently healthy individuals who belong to different BMI groups and the potential predictors remain to be investigated. In this study, we aim to determine the prevalence of insulin resistance in non-obese healthy young females from Qatar and identify the best predictors of insulin resistance for diagnostic/therapeutic implications before the onset of diabetes.

## 2. Methods

### 2.1. Participants

One hundred and fifty healthy young (22.4 ± 4.6 years old) females with a range of BMI (24.3 ± 5.3 kg/m^2^) were recruited at Qatar University campus. Students were selected through posters and advertisements in social media. Protocols were approved by the Institutional Review Board of Qatar University (QU-IRB 383-A/14) and were carried out in accordance with the Declaration of Helsinki as revised in 2008. A consent form to participate in the study was obtained from each participant. The study targeted university students to investigate the prevalence and mediators of insulin resistance in age-matched groups.

### 2.2. Anthropometric and Biochemical Measurements

Weight and body composition were determined using body composition analyzer (Inbody 720, Inbody, Cerritos, CA, USA). Height was measured by a stadiometer (Seca, Hamburg, Germany). Waist circumference was determined using a meter tape (Seca, Hamburg, Germany). Blood samples were obtained after overnight fasting from each participant. Fasting glucose, total cholesterol (TC), triglycerides (TG) and high-density lipoprotein cholesterol (HDL-C) were assayed by routine automated laboratory methods at the clinical chemistry laboratories at Hamad Medical Corporation (HMC) using Hitachi-917 (Gmbh Diagnostic, Mannheim, Germany). Insulin was determined by Elisa kit (Mercodia Insulin ELISA, Uppsala, Sweden), IL-6 (Interleukin-6 High Sensitivity Human ELISA Kit, Abcam, USA), adipokines ELISA Kits (R&D Systems, Minneapolis, USA) and TNF-alpha (Human TNF-alpha Quantikine ELISA Kit, R&D Systems, Minneapolis, USA). Insulin resistance was calculated by HOMA-IR [1] with 1.85 as a cut-off point (75th percentile) [2], accordingly samples were divided into insulin sensitive (IS) and IR groups.

### 2.3. Statistical Analysis

Comparisons were performed using a *t* test, Wilcoxon–Mann–Whitney test, one-way ANOVA or stepwise linear regression model as appropriate using IBM SPSS statistics 21 (Armonk, NY, USA). Non-parametric tests were used for comparing ordinal or non-normal variables. Orthogonal partial least square discriminant analysis (OPLS-DA) was employed to identify components that separate insulin sensitive (IS) and IR groups. The model was run using SIMCA 14 including samples with less than 50% missing metabolite values. Linear models for association analysis were run using the R statistical package version 2.14, (www.r-project.org/). A receiver operating characteristic (ROC) analysis was conducted using SPSS statistics 21 to evaluate the ability of the identified predictors of HOMA-IR described in Table 3 to correctly discriminate between IR and IS groups. The ROC curves for the established model were made and the overall diagnostic accuracy was quantified using the area under the ROC curve (AUC). The optimal cut-off points were determined by the Youden’s index, and the corresponding sensitivity and specificity were calculated. Significance was defined as *p*  ≤  0.05.

## 3. Results

### 3.1. General Characteristics and Prevalence of Insulin Resistance in Age-Matched Individuals with Different BMI Groups

Participants were dichotomized into IS and IR based on HOMA-IR. General characteristics of the study population are shown in Table 1. Results indicated that IR participants had higher BMI, body fat mass, % fat mass, waist circumference and waste to height ratio (WHtR) than age-matched IS counterparts. There was no significant difference in blood glucose between IR and IS. The prevalence of insulin resistance was 22.7% among the study population and it increased with BMI. The lowest prevalence was observed among underweight participants (7.1%) and the highest rate was for obese (45%). In all groups, the difference in HOMA-IR was mostly related to insulin levels as they had similar glucose levels. Furthermore, IR participants showed higher TG (0.96 ± 0.43 vs. 0.74 ± 0.23, *p* < 0.001), TG/HDL ratio (0.82 ± 0.54 vs. 0.56 ± 0.25, *p* < 0.001), LDL oxidase (23.5 ± 4.7 vs. 21.2 ± 4.4, *p* = 0.03) but lower adiponectin (21.1 ± 7.1 vs. 16.6 ± 6.3, *p* = 0.01), than their IS counterparts. When comparing lipids, and adipokines between IS and IR in various BMI groups (Table 2), TG was found higher in normal weight IR compared to IS counterparts, whereas Apolipoprotein A (ApoA) was lower in normal weight IR compared to normal weight IS. Adiponectin was higher in overweight IS compared to overweight IR. TG/HDL ratio was higher in obese IR compared to obese IS. IL-6 was lower in normal weight IR compared to normal weight IS, so was TNF-alpha in overweight IR compared to overweight IS. None of the other lipids or adipokines were significantly different between the IS and IR in BMI groups (Table 2). As expected, the percentage of participants with fasting blood glucose above 6.1 mM increased with BMI (0% in underweight, 2.4% in normal weight, 2.9% in overweight and 5% in obese). In addition, the percentage of women with HDL levels lower than 2 mM were highest in overweight (22.9%) and obese (35%) compared to underweight (0%) and normal weight (7.3%). Surprisingly, the percentage of women with total cholesterol levels above 5.2 mM was higher in underweight (14.3%) and normal weight (14.6%) compared to overweight (2.9%) and obese (10%).

### 3.2. Multivariate Analysis of Mediators of the Metabolic Syndrome

**Figure 1:**
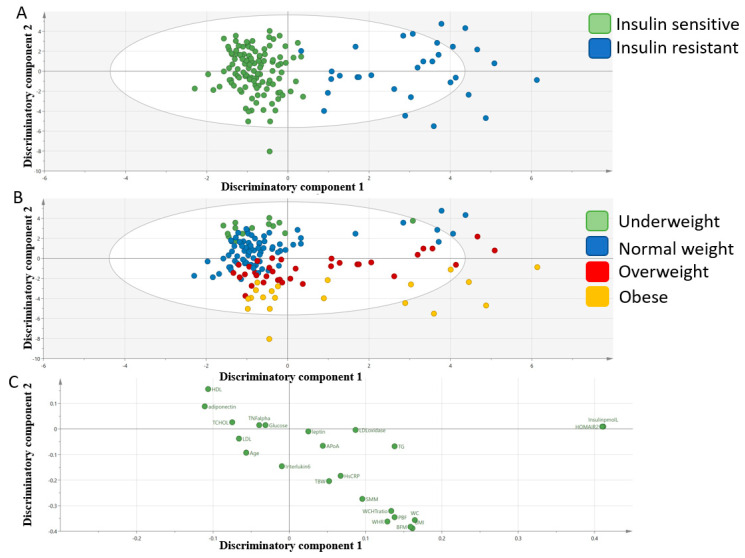
Orthogonal partial least square discriminate analysis (OPLS-DA) model comparing mediators of metabolic disease in IS and IR participants. (**A**). A score plot showing the class-discriminatory component 1 (x-axis) versus orthogonal component (y-axis). (**B**). An updated score plot that reveals that the orthogonal component (y-axis) mostly represent BMI groups (underweight, normal weight, overweight and obese). (**C**). The corresponding loading plot showing mediators of metabolic syndrome at either ends of the discriminatory components along the x-axis and y-axis.

An orthogonal partial least square discriminate analysis (OPLS-DA) comparing mediators of the metabolic syndrome between IS and IR subjects was used for ease of visualization. The model revealed two class-discriminatory components accounting for 61.5% of the variation in the data due to participants’ group (Figure 1). The score plot in Figure 1A shows an x-axis separating IS and IR groups and y-axis that clearly separated the BMI groups. The loading plot in Figure 1B shows mediators of metabolic syndrome that were responsible for separating the IS and IR groups on the x-axis including insulin and HOMA-IR on the IR end and HDL and adiponectin on the IS end. The loading plot also shows mediators of metabolic syndrome that best separated BMI groups including HDL and adiponectin in the underweight end and body mass index (BMI), waist to hip ratio (WHR), body fat percentage BFP and waist circumference (WC) in the obese end.

### 3.3. Mediators of Insulin Resistance

**Figure 2:**
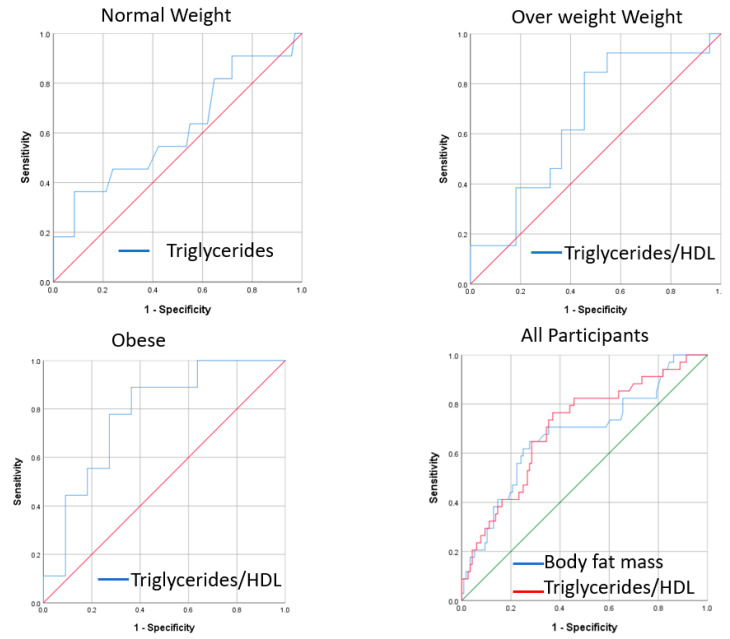
ROC curve for triglycerides, triglycerides/HDL and body fat mass as a predictor for the HOMA-IR index in normal, overweight/obese and all participants.

A generalized linear model was performed to identify mediators that best explain variance in HOMA-IR irrespective of BMI. As expected, the model identified TG, IL-6, adiponectin, HDL and TNF-alpha as top variables explaining HOMA-IR with TG exhibiting highest impact (66.4%) whereas TNF-alpha showing the least (0.8%; Table 3). A follow-up stepwise linear regression model identified TG/HDL ratio and free fat mass to best predict insulin resistance irrespective of BMI. Within the BMI groups, TG, TG/HDL and TG/HDL + IL-6 were the best predictors of insulin resistance in normal weight, overweight and obese subjects respectively (Table 4, Figure 2).

## 4. Discussion

Pre-diabetes represents a transitional state of hyperglycemia as resistance to insulin becomes above normal but below the diabetes threshold. Although criteria used for diagnosis of pre-diabetes vary among different studies, pre-diabetes constitutes an increased risk for developing diabetes at an annual rate of 5%–10% [4]. The association between pre-diabetes and various diabetes complications such as early nephropathy, small fiber neuropathy, early retinopathy and a risk of macrovascular disease is well established [5]. Lifestyle interventions were shown to reduce diabetes incidents 40%–70% in adults with pre-diabetes [4]. Results from a 12-month life style intervention program targeting obese with prediabetes revealed that 37% of participants did not show any criteria of prediabetes after the intervention. The dietary intervention consisted of lower energy dense foods and foods with high glycemic index [6]. A recent meta-analysis reported a 36% reduction in the risk of developing diabetes among prediabetes enrolled in life style intervention [3]. Despite this information, systematic evaluation of pre-diabetes in the general population is still lacking.

The prevalence of T2D is particularly high among certain ethnicities such as South Asians, Middle Eastern and Africans at a lower BMI and younger age compared to Caucasians [7]. The state of Qatar has suffered from a diabetes epidemic in the past few decades. This epidemic was attributed mainly to the high prevalence of obesity that resulted from a sudden transition from active to sedentary lifestyle following the discovery of gas and oil [8]. Although the rise in insulin resistance in non-obese individuals could also explain the high prevalence of diabetes in this population, it has not yet been addressed. In this study, prevalence of insulin resistance measured by HOMA-IR was investigated in age-matched young females from Qatar who belong to different BMI groups. The novel findings reported here indicate a high prevalence of insulin resistance prior to onset of obesity and identifies predictors of insulin resistance in different BMI groups.

Several studies have identified IR individuals using the gold-standard hyperinsulinemic–euglycemic clamp technique [9,10]. However, this technique is invasive, expensive as well as time consuming. Therefore, other methods have been used to identify IR individuals, such as the Matsuda index [11], an oral glucose tolerance test derived index and the HOMA-IR [12], a fasting surrogate index. The latter index has been also combined with clinical criteria to identify IR individuals [13]. Furthermore, several studies have defined IR individuals based on the extremes of insulin (that is, upper quartiles or lower tertiles) [1,14]. Other factors such as age and sex were also reported to affect IR prevalence. For example, the prevalence of IR individuals is lower in younger individuals of the metabolically healthy obese Italian individuals particularity in women [15]. However, caution should be taken when considering the prevalence of IS subjects in these studies as numbers could be simply a reflection of the predefined selection criteria and the stringency used to detail the phenotype. Despite its crude nature, HOMA-IR was used in this study to dichotomize participants into IS and IR using 1.85 as a cut-off point (75th percentile) [2], because of its non-invasive nature and better compliance.

In this study, there was a positive correlation between HOMA-IR and BMI (R^2^ = 0.3, *p* = 0.001). Although expected, the increase in insulin resistance with BMI was surprisingly high in the overweight (37.1%) and obese (45%) groups. Previous studies in other ethnicities have reported that around 25% of overweight individuals were insulin resistant [16,17,18]. The high prevalence of insulin resistance seen in this population compared to previous reports may partially explain the high incidents of diabetes [19]. Previous studies have indicated that insulin resistance could predict up to 80% of increased risk of diabetes among non-obese persons [18], which in this population may suggest that almost 30% of overweight participants could develop T2DM. This high risk of diabetes among this seemingly healthy young group constitutes a challenge to the local authorities as it poses a high burden on health services because of associated morbidities and all-cause mortality including risk of cardiovascular disease [20]. Hence, identifying best predictors of insulin resistance could provide important tools for screening programs aiming at targeted intervention before the onset of diabetes.

Our data indicated that variance in overall HOMA-IR was mainly explained by TG and IL-6. However, when considering different BMI groups, predictors of insulin resistance varied. The main predictor of insulin resistance in the normal weight group was TG, although the predictive power was lower than TG/HDL in other BMI groups as suggested by AUC in ROC. On the other hand, TG/HDL was the best predictor of insulin resistance in overweight and obese groups. Similar to our findings, previous reports have indicated that lean individuals with non-alcoholic fatty liver disease (NAFLD) have higher HOMA-IR and lower serum adiponectin compared to overweight-without-NAFLD with TG content being the most important determinant of insulin resistance [21]. Previous studies have also indicated that TG/HDL and TG were significantly associated with insulin resistance in normal-weight and overweight/obese Chinese women [22] and Taiwanese adults [23]. Other studies have also shown that risk of metabolic syndrome was incrementally associated with TG/HDL with no consensual cut-off values [24]. Our results confirmed the relationship between lipid and carbohydrate metabolism disorders. In this model, hyperinsulinemia can develop against pre-existing lipid metabolism disorders where lipid accumulation in skeletal muscle and liver could result from elevated delivery and synthesis of fatty acids in these tissues when energy intake exceeds the storage capacity of adipose tissue storage [25].

### Study Limitations

This was an observational study with a relatively small number of participants, hence, results need to be confirmed in a larger cohort. Additionally, the presence of the non-obese IR phenotype at a young age confirms that models based solely on BMI may not be sensitive enough to detect insulin resistance in this group. Some studies even indicated that certain non-obese individuals could be considered obese based on the percentage of their body fat. [26,27], although this was not the case in our young female group, perhaps because of ethnic or age differences. However, it is possible that a subset of the non-obese IR individuals exhibit greater intramuscular fat deposition as previously reported [28].

## 5. Conclusions

Pre-diabetes is a state of intermediate hyperglycemia. While there are several controversies about the diagnosis of pre-diabetes, it remains an at-risk state for the development of diabetes. Several adverse health outcomes have been associated with pre-diabetes. In this study, we highlighted the high prevalence of insulin resistance in overweight young females from Qatar and identified TG and TG/HDL ratio to be predict insulin resistance in this group. Preventive strategies will require addressing modifiable risk behaviors, including lack of physical activity and dietary intake, aiming at ameliorating risk of T2DM in this population.

## Figures and Tables

**Table 1:** General characteristics of study participants divided into underweight, normal weight, overweight and obese insulin sensitive (IS) and insulin resistant (IR) participants.

Groups		Combined			Under Weight		Normal Weight			Overweight			Obese		
		(116 IS and 34 IR)			(13 IS and 1 IR)		(71 IS and 11 IR)			(22 IS and 13 IR)			(11 IS and 9 IR)		
Characteristics		(IR Prevalence 22.7%)			(IR Prevalence 7.1%)		(IR Prevalence 13.4%)			(IR Prevalence 37.1%)			(IR Prevalence 45%)		
		Mean	SD	*p* Value	Mean	SD	Mean	SD	*p* Value	Mean	SD	*p* Value	Mean	SD	*p* Value
Age (years)	IS	22.5	4.9	0.449	21.4	1.7	22.4	5.1	0.938	23.4	5.6	0.551	23.1	4.8	0.237
	IR	21.8	3.6		18	N/A	22.3	5.3		22.4	3.5		21.1	1.4	
BMI (Kg/m^2^)	IS	23.5	4.6	<0.001	17.3	0.9	21.9	2	0.946	27.1	1.3	0.424	33.2	3.2	0.184
	IR	28	5.8		18	N/A	21.8	1.5		27.5	1.6		35.2	3.3	
Body fat mass	IS	21.5	8.8	<0.001	10.3	2.5	18.6	4.2	0.998	27.8	4.1	0.628	40	6	0.391
	IR	29.5	11.1		12.1	N/A	18.6	4.9		28.4	3.8		42.7	8.1	
WC (cm)	IS	81.6	10	<0.001	70.3	6.3	78.8	7.2	0.694	89.4	7.1	0.491	95.9	7.3	0.013
	IR	90.6	13		87	N/A	77.8	8.9		87.9	4		106.3	9.5	
Total body water	IS	28.8	5.6	0.366	24.7	2	28.5	6.5	0.048	30.4	2.9	0.171	31.9	3	0.163
	IR	29.7	4.1		27.7	N/A	26.3	1.8		29	2.8		34.1	3.9	
Free fat mass	IS	20.7	3.1	0.037	17.8	1.7	20.1	2.8	0.309	22.6	2.4	0.216	23.7	2.4	0.149
	IR	22	3.4		20.2	N/A	19.1	1.5		21.5	2.2		25.5	3.2	
% body fat	IS	34.6	7.8	<0.001	23.2	4.1	32.9	5.3	0.713	39.9	4.2	0.236	47.8	2.8	0.696
	IR	41	7.6		24.2	N/A	33.7	4.8		41.7	4.1		48.4	4.2	
WHtR	IS	0.5	0.1	<0.001	0.4	0	0.5	0.1	0.811	0.6	0	0.866	0.6	0	0.029
	IR	0.6	0.1		0.5	N/A	0.5	0.1		0.6	0		0.7	0.1	
Glucose (mmol/L)	IS	5	0.4	0.581	5	0.3	5	0.4	0.912	5	0.4	0.059	4.9	0.5	0.259
	IR	5	0.6		5.1	N/A	5.1	0.4		4.7	0.5		5.2	0.7	
Insulin (pmol/L)	IS	2.8	2.2	<0.001	2.4	2.5	2.4	2.1	<0.001	4	2.2	<0.001	3.2	1.8	<0.001
	IR	20.3	7.8		20.9	N/A	20.8	7.3		18.9	7.2		21.9	9.8	
HOMA-IR	IS	0.6	0.5	<0.001	0.5	0.5	0.5	0.5	<0.001	0.9	0.5	<0.001	0.7	0.4	<0.001
	IR	4.5	1.7		4.7	N/A	4.6	1.5		4.1	1.8		4.9	2	

Results are presented as mean and SD. BMI body mass index, WC waist circumference, WHtR waist to height ratio, HOMA-IR homeostatic model assessment of insulin resistance. Differences between IS and IR were tested by an independent sample *t* test (normally distributed variables) or Mann–Whitney U (variables with skewed distribution) test. A *p*-value significance level of 0.05 was used.

**Table 2:** Profiles of lipids and adipokines in underweight, normal weight, overweight and obese insulin sensitive (IS) and insulin resistant (IR) participants.

Groups		Combined			Under Weight		Normal Weight			Overweight			Obese		
Characteristics		Mean	SD	*p* Value	Mean	SD	Mean	SD	*p* Value	Mean	SD	*p* Value	Mean	SD	*p* Value
LDL (mmol/L)	IS	2.4	0.7	0.123	2.2	0.7	2.5	0.7	0.102	2.4	0.4	0.486	2.4	0.9	0.998
	IR	2.2	0.5		1.8	N/A	2.1	0.5		2.2	0.5		2.4	0.6	
HDL (mmol/L)	IS	1.4	0.3	0.049	1.6	0.4	1.5	0.4	0.867	1.3	0.3	0.581	1.2	0.2	0.427
	IR	1.3	0.4		1.5	N/A	1.4	0.3		1.2	0.4		1.1	0.2	
Triglyceride (mmol/L)	IS	0.7	0.2	<0.001	0.7	0.2	0.7	0.2	0.027	0.8	0.2	0.159	0.8	0.3	0.115
	IR	1.0	0.4		0.7	N/A	0.9	0.4		1.0	0.5		1.0	0.3	
Cholesterol (mmol/L)	IS	4.2	0.9	0.147	4.2	1.0	4.3	0.9	0.288	4.0	0.6	0.694	3.9	1.0	0.972
	IR	3.9	0.7		3.6	N/A	4.0	0.8		3.9	0.7		3.9	0.8	
APoA (g/L)	IS	0.6	0.7	0.494	0.6	0.5	0.5	0.6	0.204	0.9	0.9	0.673	0.7	0.4	0.372
	IR	0.7	0.7		0.4	N/A	0.3	0.2		1.0	1.0		0.9	0.6	
LDL oxidase	IS	21.2	4.4	0.031	20.4	3.2	21.8	4.0	0.625	20.1	6.1	0.218	20.6	3.1	0.174
	IR	23.5	4.7		27.2	N/A	22.6	3.2		22.7	3.4		25.2	7.7	
Leptin (ng/mL)	IS	3.4	1.5	0.262	4.2	1.6	3.6	1.5	0.169	2.5	1.0	0.077	4.1	1.9	0.987
	IR	3.8	1.4		4.8	N/A	4.3	1.1		3.1	1.2		4.1	1.7	
Adiponectin (ng/mL)	IS	21.1	7.1	0.01	23.9	6.7	22.8	7.4	0.991	20.2	7.0	0.03	17.8	6.2	0.952
	IR	16.6	6.3		N/A	N/A	22.7	1.0		14.8	5.6		17.6	7.0	
CRP (mg/L)	IS	2.4	2.7	0.077	2.1	2.6	1.7	2.3	0.744	3.4	2.7	0.957	4.5	3.9	0.726
	IR	3.3	3.0		1.2	N/A	2.0	2.8		3.4	3.0		5.1	2.9	
IL-6 (pg/mL)	IS	3.0	3.5	0.758	2.9	1.7	2.7	1.9	0.167	2.7	1.0	0.147	6.2	9.8	0.359
	IR	2.8	1.2		4.6	N/A	1.9	0.7		3.3	1.3		3.1	0.9	
TNFalpha (pg/mL)	IS	119.8	273.2	0.665	33.2	13.8	148.8	314.7	0.582	127.5	290.9	0.594	42.9	47.7	0.332
	IR	92.3	203.9		N/A	N/A	22.5	5.7		76.4	199.1		129.2	238.1	

Results are presented as mean and SD. Low density lipoprotein (LDL), High density lipoprotein (HDL), Apo lipoprotein A (ApoA), Differences between IS and IR were tested by an independent sample *t* test (normally distributed variables) or Mann–Whitney U (variables with skewed distribution) test. A *p*-value significance level of 0.05 was used.

**Table 3:** Variables explaining HOMA-IR by generalized linear model.

Variables Explaining HOMA-IR	Importance	*p* Value
**Triglycerides**	0.67	<0.001
**Interleukin-6**	0.29	<0.001
**Adiponectin**	0.03	<0.001
**HDL**	0.01	<0.001
**TNF-alpha**	0.008	<0.001

**Table 4:** Predictors of HOMA-IR in normal weight, overweight and obese groups by stepwise linear regression followed by the receiver operating characteristic (ROC) curve.

Group	Predictor	Adjusted	Std. Error of the Estimate	*p* Value	Area Under Curve	(95% CI)
		R Square				
**Normal weight**	TG	0.23	1.2	0.01	0.61	(0.41–0.81)
**Overweight**	TG/HDL	0.22	1.3	0.01	0.66	(0.47–0.85)
**Obese**	TG/HDL	0.44	2	0.01	0.78	(0.57–0.99)
**All groups**	TG/HDL	0.31	1.4	<0.001	0.7	(0.60–0.80)
	TG/HDL and Free fat mass	0.37	1.4	0.02	0.68	(0.57–0.79)
